# Micronucleus Frequency in Exfoliated Buccal Cells of Children Living in an Industrialized Area of Apulia (Italy)

**DOI:** 10.3390/ijerph17041208

**Published:** 2020-02-13

**Authors:** Alessandra Panico, Tiziana Grassi, Francesco Bagordo, Adele Idolo, Francesca Serio, Maria Rosaria Tumolo, Mattia De Giorgi, Marcello Guido, Maria Tutino, Antonella De Donno

**Affiliations:** 1 Department of Biological and Environmental Sciences and Technologies, University of Salento, 73100 Lecce, Italy; alessandra.panico@unisalento.it (A.P.); tiziana.grassi@unisalento.it (T.G.); adele.idolo@unisalento.it (A.I.); francesca.serio@unisalento.it (F.S.); mattia.degiorgi@unisalento.it (M.D.G.); marcello.guido@unisalento.it (M.G.); antonella.dedonno@unisalento.it (A.D.D.); 2 Institute for Research on Population and Social Policies, National Research Council (IRPPS-CNR), 72100 Brindisi, Italy; mariarosaria.tumolo@irpps.cnr.it; 3 Institute of Clinical Physiology (CNR-IFC), 73100 Lecce, Italy; 4 Regional Agency for Environmental Protection (ARPA Puglia), 70126 Bari, Italy; m.tutino@arpa.puglia.it

**Keywords:** micronuclei, buccal micronucleus cytome assay, children, environmental exposure, lifestyle, air pollution, quasi-ultrafine particles, PJS Study

## Abstract

Micronuclei (MN) are biomarkers of early biological effect often used for detecting DNA damage in human population exposed to genotoxic agents. The aim of this study was to evaluate the frequency of MN in exfoliated buccal cells of children living in an industrialized (impacted) area compared with that found in children living in a control area without significant anthropogenic impacts. A total of 462 6–8-year-old children (206 in the impacted area, 256 in the control area) attending primary school were enrolled. A questionnaire was administered to the parents of the recruited children to obtain information about personal data, lifestyles, and food habits of their children. Atmospheric particulate fractions were collected near the involved schools to assess the level of environmental exposure of the children. The presence of MN was highlighted in 68.4% of children living in the impacted area with a mean MN frequency of 0.66‰ ± 0.61‰. MN positivity and frequency were significantly lower in the control area (37.1% and 0.27‰ ± 0.43‰, respectively). The frequency of MN was positively associated with quasi-ultrafine particulate matter (PM_0.5_), traffic near the home, and consuming barbecued food; while adherence to the Mediterranean diet and practicing sport were negatively associated.

## 1. Introduction

Molecular epidemiology is an innovative approach to early assessment of the health risks due to environmental exposure. It allows for the direct measurement of biological effects caused by the contact with toxic substances by means of biomarkers [1,2].

In the last decades, micronuclei (MN) have been widely used in molecular epidemiologic studies [3,4,5,6,7,8]. They are considered as biomarkers of early biological effect that are formed in the cells due to alterations of the chromosomal structure and oxidative stress attributable to various factors, among which is environmental exposure [9,10,11].

Particulate matter (PM), and, in particular, PM_10_, is well known to have toxic and carcinogenic effects on the human population [12]. However, in the most recent studies investigating the frequency of MN in populations exposed to atmospheric pollution, the effects of fine particulates (PM_2.5_–PM_1_), quasi ultrafine particulates (PM_0.5_–PM_0.3_), and ultrafine particulates (PM_0.1_) have been studied more extensively than PM_10_ [13,14].

MN can be investigated in various organs, tissues, and body fluids, depending on the causal factors (genetic, environmental, pathological, etc.) to be evaluated.

In recent years, exfoliated cells from buccal mucosa have been increasingly used to detect MN frequency related to atmospheric exposure [1,10,15,16]. Buccal mucosa (BM) is a very sensitive tissue, directly exposed to airborne pollutants. It is easily accessible for sampling cells in a minimally invasive manner that does not cause undue stress to study subjects, especially when they are children [17]. Moreover, the evaluation of genotoxic end-points in rapidly dividing cells (such as BM cells) allows for the assessment of cytogenetic damage (i.e., MN) without establishing the ex vivo cell replication step typically required by classical metaphase or interphase analyses (e.g., the cytokinesis-block MN assay in binucleated lymphocytes) [18]. Nowadays, the human buccal micronucleus cytome (BMCyt) assay is one of the most widely used techniques to measure genetic damage in studies on human population exposed to airborne pollutants [19,20].

The difficulties in the large-scale application of this biomarker are the variability of the micronuclei frequency due to different individual and behavioral factors and the identification of a normal range within which this frequency can vary. In fact, the MN frequency could be modulated by other conditions able to affect genome stability, such as genetic factors, lifestyle, and health status, which vary individually as well as over time and space.

In the literature, numerous studies conducted on both adults and children have provided evidence regarding the positive correlation between the frequency of early-effect biomarkers and factors such as obesity [21,22], respiratory diseases [23,24], residence in areas with high traffic levels [25], exposure to passive smoking [26,27], and inhalation or ingestion of toxic compounds formed during cooking with certain methods (grilling, on the griddle, frying, toasting) [28,29,30]. In contrast, a high level of parental education [31,32] and intake of antioxidant substances and vitamins in the diet [19,33,34] could be negatively related to the frequency of MN. Moderate physical exercise is generally associated with lower MN in peripheral blood lymphocytes [35].

Therefore, it is necessary to apply this biomarker in relation to different confounding factors and to compare the MN frequency detected in anthropized areas with that detected in areas where the anthropogenic impact is zero or negligible.

In the Apulia region (Italy), some areas with high environmental criticality due to the presence of important industrial activities have been identified. In recent years, the industrial area of Brindisi has had an important qualitative and quantitative development, which has caused the release of new polluting substances. This area is located near some cities and could adversely affect the health of the citizens who live there. The studies carried out to date [36] took into consideration only the epidemiological data and found an increase in mortality for some cancers and cardiovascular diseases, mainly linked to older emissions. However, these data, by their nature, did not allow for the establishment of any causal links between the morbid events and the recent environmental framework. Therefore, epidemiological studies on existing cohorts should be continued and supplemented with molecular epidemiology research. For this reason, the Apulia Region Council has promoted a study to determine the risk level of people living close to industrialized areas by detecting early risk indicators.

The aim of this work was to evaluate the frequency of MN in the exfoliated buccal cells (EBC) of children living in an area with a high industrial impact compared with that found in children living in an area without significant anthropogenic impacts. The environmental factors, with particular reference to the PM exposure and lifestyle aspects associated with the variability in the frequency of MN, were also evaluated.

## 2. Materials and Methods 

### 2.1. Study Design

This study is part of the Jonico-Salentino Project (PJS), a research project funded by the Apulia Region aimed at identifying the risk profiles of people living in the provinces of Brindisi, Lecce, and Taranto on the basis of their actual exposure to any polluting sources. The exposure risk assessment methodologies comprised both official methods and innovative ones, including the use of new outcome indicators obtained from human biomonitoring surveys, such as those assessed in the present study.

The study was performed following the protocol of the MAPEC_LIFE (Monitoring Air Pollution Effects on Children for Supporting Public Health Policy) study [16]. It envisaged (a) recruitment of primary schoolchildren in two areas of Salento Peninsula (an “impacted area” and a “control area”); (b) questionnaire administration to parents to obtain information about lifestyle of their children; (c) sampling of EBC of the children; (d) assessment of genotoxic damage in the sampled cells by BMCyt assay; (e) atmospheric monitoring near the schools involved in order to determine the concentrations of particulate matter (PM); and (f) data analysis to identify any association between DNA damage and environmental or individual factors. The monitoring activities were performed during the same period (the month of May) in order to minimize the effect due to seasonal variability.

### 2.2. Study Areas

**Figure 1:**
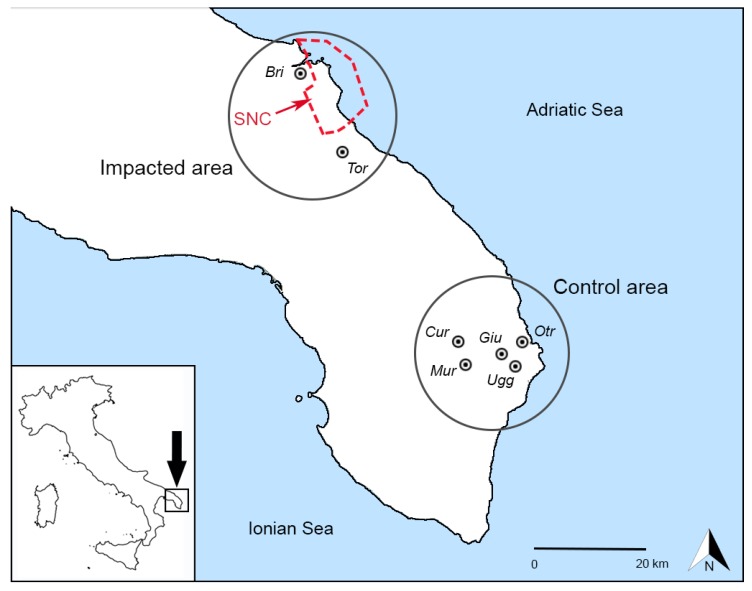
Map of the study area (SNC: Site of National Concern; Bri: Brindisi; Cur: Cursi; Giu: Giurdignano; Mur: Muro Leccese; Otr: Otranto; Tor: Torchiarolo; Ugg: Uggiano La Chiesa).

The study was conducted in the years 2017–2018 in two different areas of the Salento peninsula (Figure 1):

1. An impacted area affected by industrial activity;
2. A control area without significant anthropic activity.

The impacted area is located in the northeast of Salento (the Southern part of Apulia) and includes the Municipalities of Brindisi and Torchiarolo. Brindisi is a city with a total population of 87,820 inhabitants and a 6–8-year-old population of 2451 children [37]. Torchiarolo is a small village with a total population of 5459 inhabitants and a 6–8-year-old population of 145 children [36]. Between these two towns there is an industrial area with chemical plants and electric power production from coal. This zone is included in a larger area that was declared in 1998 as a Site of National Concern (SNC) (Legislative Decree 426/98) since it was characterized by a significant anthropic contamination of environmental matrices with high levels of toxic pollutants and strong impact on the environment in terms of health and ecological risk. Brindisi SNC covers an area of approximately 5700 hectares of land and 5600 hectares of sea and includes, in addition to the industrial area, also the whole port and a strip of coastline of about 30 km^2^. Brindisi and Torchiarolo are located respectively in the northwest and southeast of the SNC area. During the year 2017, the prevailing wind in the area was from northwest, such as during the research period, and the average wind speed was 2.25 ± 1.38 m/s, while during the research period it was 2.41 ± 1.45 m/s [38].

The control area is located about 50 km south-east from the impacted area and includes five small villages (Cursi, Giurdignano, Muro Leccese, Otranto, and Uggiano La Chiesa) with a total population of 21,177 inhabitants and a 6–8-year-old population of 518 children [37], whose characteristics were previously reported [39]. This area has no evident industrial impacts and is characterized by a predominantly agricultural economy [40].

### 2.3. Recruitment

In each small village, the only one primary school was selected. In the town of Brindisi, three primary schools were selected; they were located in the center of the urban area, far from punctual sources of electromagnetic, radioactive, or industrial pollution, with a high number of pupils. In each school, meetings were held with teachers and parents of children attending first, second, and third year classes in order to explain the study and promote participation. Subsequently, a project parcel was distributed to the parents. This parcel contained a) a fact sheet with information about the project, its objectives and methods; b) the informed consent form for the children’s parents’ approval; c) the assent form for the children’s approval. After a few days, the parents’ consent and children’s assent forms were gathered and checked in order to verify their approval. Participation in the study was voluntary.

### 2.4. Questionnaire Administration

In the weeks preceding the biological and environmental sampling, the parents who had agreed to participate in the study were asked to fill in a questionnaire (Document S1) developed by Zani et al. [41]. It was subdivided into various sections: criteria for exclusion from the study (age below six years or equal to/above nine, residence in cities other than those involved in the study, the presence of serious illness, exposure to radiotherapy or chemotherapy in the 12 months preceding the investigation, exposure to radiographic testing in the month preceding the investigation, use of dental braces); the child’s personal information (gender, date, nation of birth); the child’s health status (respiratory problems and use of medicine); domestic environment (intensity of traffic near the home and the school, including heavy goods vehicles, fuel used for heating and cooking, presence of gas boilers, stoves and fireplaces inside the dwelling, presence of smokers inside the dwelling, cooking on the griddle/barbecue, use of solvents for hobbies); the child’s lifestyle (sports, outdoor exercise, frequent staying in the kitchen during the cooking of food); the parents’ characteristics (nation of birth, level of education, occupation, smoking habits); and the child’s eating habits (considering also the consumption of barbecued, griddled, fried, toasted, or smoked foods whose cooking methods are considered “risky” for the production of toxic substances). The dietary section was based on the questionnaire used in the ARCA (Italian acronym: diet in the Campania Region) project [42], modified with the addition of some information regarding breakfast and weekly fast food frequency.

All the questionnaires were first checked in order to eliminate incomplete and incorrect ones. In addition, the questionnaires reporting positive responses regarding the exclusion criteria were excluded from the study. Finally, an alphanumeric code was assigned to each valid questionnaire, which also unequivocally identified the biological samples of the children.

The age of each child was measured by calculating the time interval between the date of birth given on the questionnaire and the date of completion of the questionnaire. The different items listed in the food frequency section of the questionnaire were used to determine whether the child follows the Mediterranean diet (MD). The adherence of children to MD was evaluated with the “Mediterranean Diet Quality Index for children and adolescents” (KIDMED) [43], based on the principles sustaining Mediterranean dietary patterns as well as those that undermine it. It includes 16 yes or no questions: those indicating a negative connotation with respect to the MD were assigned a value of −1 (going more than once a week to a fast-food restaurant, skipping breakfast, having commercially baked goods or pastries for breakfast, taking sweets and candy several times every day) and those with a positive aspect were assigned a value of +1 (taking a fruit or fruit juice every day, having a second fruit every day, having fresh or cooked vegetables regularly once a day, having fresh or cooked vegetables more than once a day, consuming fish at least 2–3 times per week, eating pulses more than once a week, consuming pasta or rice 5 or more times per week, having cereals or grains for breakfast, consuming nuts at least 2–3 times per week, using olive oil at home, having a dairy product for breakfast, taking two yoghurts and/or some cheese daily). The total score ranges from −4 to 12 and was classified into three levels: ≥8, high adherence to the MD; 4–7, moderate adherence; ≤3, low adherence.

### 2.5. Anthropometric Measurements and Biological Sampling

Children whose parents correctly completed the questionnaire were subjected to anthropometric measurements and biological sampling at school during the morning hours before break time.

The anthropometric measurements were taken according to WHO recommendations [44]. Weight and height data were used to calculate the children’s body mass index (BMI) (weight (kg)/height (m) squared). This was used in turn to assess whether the child was underweight (UW), of normal weight (NW), overweight (OW), or obese (OB) according to the cut-off points established by World Obesity Federation (WOF) [45].

The epithelial buccal cells from each child were collected by gently scraping the inner surface of both cheeks with a small-headed toothbrush as described in Idolo et al. [46]. The cells were then transferred into tubes containing Saccomanno’s fixative (50% ethanol, 2% polyethylene glycol, v/v; solution diluted in water and stored at 4 °C). The samples were stored at 4 °C and subjected to biological assay in 7 days.

### 2.6. Buccal Micronucleus Cytome (BMCyt) Assay

The BMCyt assay was performed according to the procedure described by Thomas and Fenech [47]. The cell suspensions were centrifuged (500 rpm at 4 °C for 10 minutes), washed with phosphate buffered saline (PBS) (Invitrogen Srl, Milan, Italy), filtered through a 100 μm nylon filter (Merck Spa, Milan, Italy), and centrifuged again. Cell pellets were resuspended in ice-cold PBS and fixed with ice-cold Carnoy’s fixative (methanol and glacial acetic acid 3:1) and stored at −20 °C. For each sample, two slides were prepared by smearing 100 μL of cell suspension. The slides were treated with Schiff’s reagent (Sigma-Aldrich, Milan, Italy), washed, stained with 0.2% Light Green reagent (Sigma-Aldrich, Stenheim, Germany), air-dried, and finally mounted with DePex mounting medium (VWR International PBI Srl, Milan, Italy).

**Figure 2:**
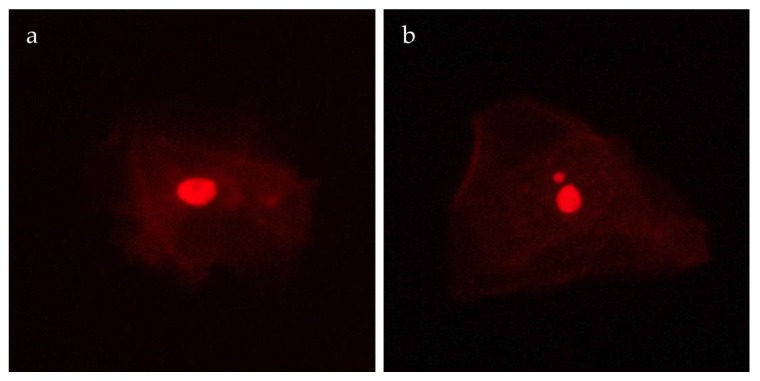
Normal differentiated cell (**a**) and cell with a micronucleus (**b**) observed by fluorescence microscope.

For each slide, 1000 differentiated cells were scored using fluorescence microscopy (Eclipse 50i, Nikon, Tokyo, Japan) according to Thomas and Fenech [47]. Micronucleated cells (Figure 2) were classified according to Bolognesi et al. [48] and their frequency was reported as number of MN in 1000 differentiated cells (‰).

### 2.7. Airborne Particulate Sampling 

Air quality was evaluated by a gravimetric method in four sites (three in Brindisi and one in Torchiarolo) located in impacted area and in five sites (one for each municipality) in the control area. A high-volume air sampler equipped with multistage cascade impactor (AirFlow PM10-HVS sampler, AMS Analitica Srl, Pesaro, Italy), compliant with UNI-EN 12341 (the Italian National Unification Body, Milan, Italy), was placed in the courtyard of each school, in an open and paved point, away from significant obstacles (walls, trees, etc.) and from punctual sources of atmospheric pollution (chimneys, vent pipes, etc.).

The sampling was performed in three consecutive 24 h periods, for a total of 72 sampling hours at a flow of 1160 L/min, by collecting atmospheric particulate fractions smaller than 10 micrometers on glass fiber membranes. The particle size fractions collected were as follows: 10.0–7.2, 7.2–3.0, 3.0–1.5, 1.5–0.95, 0.95–0.49, and <0.49 μm (PM_0.5_). All filters were pre- and post-conditioned and singularly weighed at controlled temperature and humidity, as previously reported [49].

### 2.8. Data Analysis

All the information obtained from the biological and environmental surveys as well as the questionnaire administration were entered into a Microsoft Excel database (Microsoft Corporation, Redmond, Washington, USA) and statistically processed using MedCalc Software ver. 12.3 (MedCalc Software BVBA, Ostend, Belgium).

A descriptive statistical analysis was performed for both quantitative and qualitative variables. Approximation to the normal distribution of continuous variables was evaluated using the Kolmogorov–Smirnov test. Frequencies of MN showed significant departures from the normal distribution, even after logarithmic transformation. Therefore, the differences among groups were investigated using the nonparametric Kruskal–Wallis H test. For the other continuous variables, one-way ANOVA test was used. The comparison among proportions was performed using Pearson’s χ^2^ test. Differences were considered significant at *p* < 0.05. In addition, a multivariate logistic regression analysis, with the corresponding odds ratio (OR) and 95% confidence interval (CI), were carried out to examine the possible association between personal, socioeconomic, behavioral, and environmental factors (independent variables) and positivity for the BMCyt assay (dependent variable).

### 2.9. Ethical Aspects

The study was approved by the Ethical Committee of the Local Health Authority of Brindisi (Registration Number 242/16), responsible for the impacted area, and by the Ethical Committee of the Local Health Authority of Lecce (Registration Number 15/18), responsible for the control area. Participation in the study was voluntary and no incentive was offered for this. All children’s parents received written and oral information on the study and, if agreed to, signed the informed consent form. The children also expressed their approval to participate in the study through a specially prepared assent form. All the data were gathered and analyzed in accordance with Legislative Decree 196 of 30/6/2003 ("protection of personal data”) and subsequent additions, for research purposes.

## 3. Results

### 3.1. Recruitment

The results of the recruitment and questionnaire administration activities are illustrated in Table 1. A total of 887 invitations (432 in the impacted area and 455 in the control area) were sent to the parents of children attending the first-, second-, and third-year classes in selected schools. Of these, 238 (55.1%) for the impacted area and 310 (68.1%) for the control area accepted to participate in the study, signed the consent form, and filled the questionnaire on lifestyle and eating habits of their children.

After the questionnaire check, 504 children were considered eligible, but only 462 were sampled (206 for the impacted area and 256 for the control area) because of the absence from school of some pupils during the biological monitoring. 

### 3.2. Characteristics of Children

The characteristics of the study population are reported in Table 2. All children were born in Italy (not reported in the table) and 51.9% of them were males. In the impacted area, the subjects had a mean age of 7.68 ± 0.80 years, a mean weight of 28.0 ± 7.10 kg, a mean height of 129.2 ± 7.27 cm, and a mean BMI of 16.6 ± 3.10 kg/m^2^. According to WOF cut-offs, 63.1% of the children were of normal weight, 16.0% were overweight, 9.2% were obese, while 11.7% were underweight. Concerning the health status, 29.1% of children suffered from respiratory diseases beyond the common cold and 16.0% had taken medicine in addition to common remedies such as antibiotics, antipyretics, and anti-inflammatory agents in the six months before sampling. One hundred and twenty-seven children (61.7%) regularly practiced a sport.

**Figure 3:**
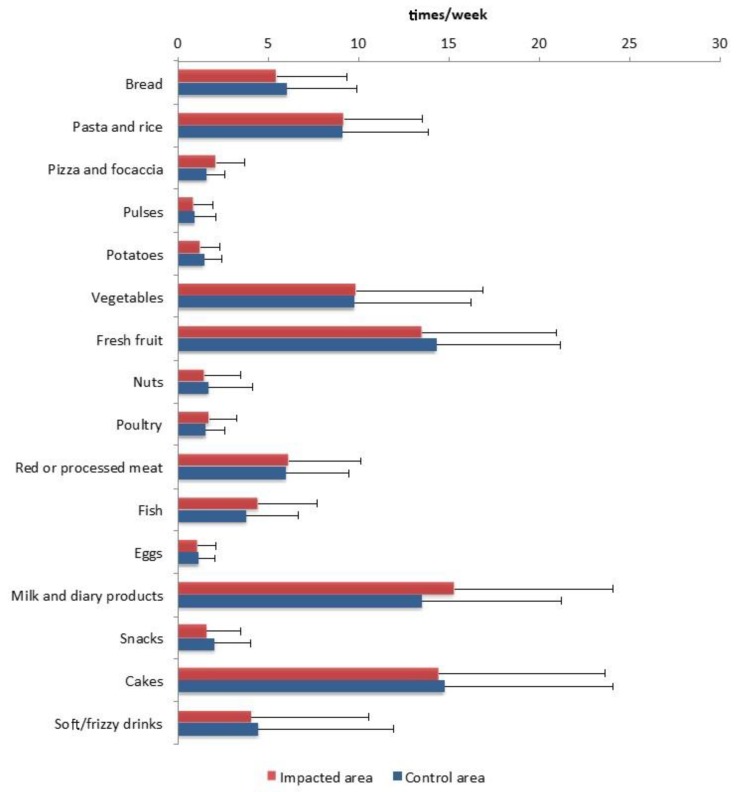
Average weekly consumption of food and beverages, grouped in food categories, by the children living in impacted and control areas.

Regarding the dietary habits (Figure 3), on average, children consumed milk and dairy products, fresh fruit, sweets, vegetables, pasta, and rice once or more than once a day; bread, red and processed meat, soft or fizzy drinks, fish, pizza, and focaccia more than once a week; potatoes, nuts, poultry, eggs, and snacks about once a day; legumes less than once a week. According to their KIDMED score (Table 2), 27.7% of children showed a high adherence to the MD, 37.9% a moderate adherence, and 34.5% a low adherence.

The children living in the control area showed similar (*p* > 0.05) characteristics, except for the prevalence of respiratory diseases beyond the common cold that was significantly lower (20.3%).

### 3.3. Characteristics of Parents

The parents of the recruited children exhibited some significantly different characteristics (*p* < 0.05) (Table 3). In both areas, most of the parents were born in Italy (93.7% of mothers and 98.1% of fathers in the impacted area; 86.7% of mothers and 90.2% of fathers in the control area), but in the control area, the prevalence of parents born out of Italy was significantly higher. In the impacted area, 29.1% of mothers and 21.8% of fathers had a college degree, showing a significant difference with the control area, where the rate of parents with a college degree was lower (19.2% of mothers and 9.4% of fathers). In the impacted area, 48.6% of mothers and 82.0% of fathers were employed, without any difference with the control area (47.8% and 86.6%, respectively). Regarding the smoking habits, 31.6% of fathers and 24.8% of mothers living in the impacted area were smokers. Mothers living in the control area showed a significantly lower attitude to smoke (13.7%).

### 3.4. Exposure Factors Related to Lifestyle

Among all children from the impacted area (Table 4), 77 (37.4%) lived in homes and 76 (36.9%) attended schools located near streets perceived as highly trafficked. Regarding the various types of heating known as causes of indoor pollutants’ release, in the month preceding the survey, fireplaces had been used in 14.3% of children’s homes (with an overall use of an average of 2.0 days per month per family) while wood or pellet stoves in 7.8% (with an overall use of an average of 1.2 days per month per family). Twenty (9.7%) children lived in homes equipped with an internal gas boiler, and 40 (19.4%) were often present in the kitchen during the food preparation. Cooking on griddle or barbecue inside the home was reported in 67.0% of the questionnaires. Forty-nine interviewed parents (23.8%) stated that their children lived with people who used to smoke inside the home and none of them reported the use of solvents for hobbies in the home (not shown in table). In the month preceding the survey, the children participating in the study had often consumed foods prepared with health-risky cooking methods due to the production of toxic substances: 89.3% of them had consumed fried foods, 49.0% barbecued foods, 62.1% food cooked on the griddle, 51.9% toasted bread, and 64.6% pizza cooked in a wood oven.

Children living in the impacted area exhibited some significant differences in exposure factors related to their homes and lifestyles compared with the children living in the control area. The latter showed a lower prevalence of residences and schools located near streets perceived as heavily trafficked (8.6% and 8.2%, respectively), living with people who used to smoke inside the house (11.3%), and consuming foods cooked on the griddle (52.0%), while there was a higher percentage of fireplace use (35.0%, with an overall use of an average of 4.9 days per month per family) and consumption of pizza cooked in a wood oven (77.7%) (Table 4).

### 3.5. Air Quality

The concentrations of the PM fractions detected using an air sampler placed in the schools’ courtyards are reported in Table 5. In the impacted area, the mean values of PM_10_ and PM_0.5_ were respectively 22.2 ± 6.8 µg/m^3^ and 9.25 ± 2.55 µg/m^3^, while in the control area, they were 11.6 ± 3.9 µg/m^3^ and 3.16 ± 1.40 µg/m^3^. The concentrations of both fractions of PM were similar (*p* > 0.05) in the two cities located at opposite sides of the SNC area.

### 3.6. BMCyt Assay

The results of BMCyt assay performed on the EBC of children participating in the study are summarized in Table 6. Overall, in the impacted area, 68.4% of sampled children were positive to the BMCyt assay with a mean MN frequency of 0.66‰ ± 0.61‰ without significantly differences (*p* > 0.05) between the two cities of the area. On the contrary, MN positivity and frequency were significantly lower in the control area (37.1% and 0.27‰ ± 0.43‰, respectively).

A multivariate logistic regression analysis was performed taking into account demographic, socio-economic, and lifestyle factors, in order to identity their possible modifier or confounder effect on MN frequency (Table 7). It showed that PM_0.5_ (OR = 1.25; 95%CI = 1.16–1.34), living near highly trafficked streets (OR = 2.12; 95%CI = 1.24–3.63), and consuming barbecued foods (OR = 1.57; 95%CI = 1.02–2.40) were positively associated with MN frequency, while adherence to the MD (OR = 0.50; 95%CI = 0.27–0.94) and practicing sports (OR = 0.64; 95%CI = 0.42–0.97) were negatively associated. The multivariate model taking into consideration fractions of PM greater than PM_0.5_ showed a lower contribution of PM_10_ (OR = 1.09; 95%CI = 1.05–1.14) to the frequency of MN (not in table).

## 4. Discussion

The frequency of markers of chromosomal DNA damage was evaluated in EBC of 462 children residing in two areas of Apulia with different anthropic impacts.

Human activities seemed to influence air quality. In fact, the environmental monitoring data showed a greater concentration of PM in the air of the impacted area than in the control one, although the values of PM_10_ had never exceeded the daily limit of 50 µg/m^3^ indicated in the European Directive 2008/50/EC on air quality. The two cities located at opposite sides of the SNC area during the study period did not show significantly different concentrations of PM. Considering that the wind mainly blew from the northwest, it is possible to hypothesize that the air quality of Torchiarolo, located southeast of the SNC area, was influenced by the activities carried out in the industrial area. Instead, the air of Brindisi probably received a lower contribution from industrial activities but was also affected by vehicular traffic, typical of a large city, and by other activities throughout the area surrounding the urban center. Since during the study period the prevailing wind direction was the same as that recorded during the whole year, it is possible to generalize this consideration for a longer period, although the effect could also depend on other meteorological factors (wind speed, humidity, temperature, precipitation, etc.) as well as the type and concentration of pollutants. Further studies are needed to better define the sources and dispersion dynamics of the various contaminants.

Recruited children highlighted a prevalence of obesity (9.7%) lower than that reported in the “Okkio alla salute” study, a national survey on the health status of Italian children, which reported an obesity prevalence of 12.6% in 3157 8–9-year-old children from Apulia [50]. In addition, Grassi et al. [51] and Censi et al. [52] reported a prevalence of obesity respectively of 17.3% in 687 6–8-year-old children living in central-southern Italy and of 13.5% in 742 8–9-year-old southern Italian children. This result could partly be explained by the level of adherence to the MD and the practice of sports, which, in these areas, seemed to be higher than that observed in previous Italian surveys [53,54,55,56].

Smoking habits of children’s parents in this study compared with national data from the “PASSI” study [57] showed that, in general, the fathers involved smoked (31.5%) slightly more than Italian men overall (29.9%) while the mothers smoked (18.7%) less than Italian women overall (21.6%). However, in the impacted area, the mothers registered a higher level of smoking habits (24.8%) than the national average.

The children’s general characteristics were similar between the two groups, with differences only in some factors. In particular, the prevalence of children suffering from respiratory diseases beyond the common cold registered in the impacted area was higher than in the control area. This evidence has been widely discussed in several studies, which proved a higher incidence of respiratory diseases in children exposed to high levels of PM [58,59,60]. Other differences between the two areas were the residence in highly trafficked areas, the exposure to passive smoking (higher in the impacted area), and to indoor pollution caused by heating (fireplaces) (higher in the control area).

The results of the biological survey showed a significant higher mean MN frequency in the children living in the impacted area (0.66 ± 0.61 MN‰) compared with the children living in the control area (0.27 ± 0.43 MN‰). A similar MN frequency was observed in EBC of 64 children with a mean age of 7.3 years living close to highly trafficked areas in Oakland, California (0.67 ± 1.44 MN‰) [61]. A higher MN frequency, compared with our study, was observed in 411 9-year-old children living near chipboard industries in the manufacturing district of Viadana, Italy (1.20 ± 0.90 MN‰) [62]. Two groups of Brazilian children aged ≤7 years and 8–9 years living in an urban polluted area measured respectively 1.20 ± 0.83 MN‰ and 1.33 ± 0.87 MN‰, while the correspondent controls living in a rural nonpolluted area highlighted a mean MN frequency of 0.19 ± 0.31 MN‰ and 0.29 ± 0.46 MN‰ [63], very close to the results observed in our control area. Villarini et al. [64] reported a mean MN frequency of 0.53 ± 0.61 MN‰ in 237 children living in Brescia, an Italian town located in the Po Valley, an area known to be highly polluted. Finally, 122 6–8-year-old children living in a rural area of Salento with moderate industrial activities showed a mean MN frequency of 0.49 ± 0.65 MN‰ [46].

The genotoxic effect observed in the buccal cells of the recruited children was associated with the level of environmental pollution defined by PM_0.5_ concentrations, traffic density near children’s homes, and the consumption of food grilled on wood or charcoal. On the other hand, sports activity and high adherence to the MD seem to counteract the formation of MN. In addition, smaller fractions of particulate seemed to have higher effects on MN formation than greater ones.

Our findings were consistent with other previous investigations. Numerous in vitro studies highlighted the role of ultrafine particulate in the MN onset. Feretti et al. [65] observed that PM_0.5_ collected in an industrialized town of northern Italy exhibited the highest genotoxic potential compared with greater fractions inducing an increase of MN frequency in Allium root cells. In Verma et al. [66], both PM_0.56_ and PM_0.056_ displayed MN formation in cells from clonal mouse embryo cell line (10T1/2) with greater effect as compared with larger PM fraction. However, in our study, PM_0.5_ could be considered as an indicator of atmospheric pollution from anthropic activities. Other pollutants, such as heavy metals [67], polycyclic aromatic hydrocarbons [19], benzene [68], and NO_2_ [15], could have even higher effects on MN formation.

As for vehicular traffic, diesel exhaust particulate material was recognized to induce MN formation in human bronchial epithelial cells (BEAS-2B) [69]. Some in vivo studies correlated micronuclei with residence in intense traffic areas, showing that cytogenetic damage was even higher in children than in adults [25,61].

To the best of our knowledge, there are no previous human studies proving the association between MN frequency in buccal exfoliated cells and ingestion of barbecued food. However, the presence of genotoxins such as acrylamide, polycyclic aromatic hydrocarbons, lipid peroxides, and heterocyclic amines in pyrolyzed foods was proved to induce in vitro MN formation [35]. In addition, studies on murine model established an increased MN frequency after barbecued food ingestion [70], and professional exposure of barbecue grillers to cooking fumes may enhance the MN formation in their EBC [71].

Regarding sports activity, previous studies reported a reduction of MN in peripheral blood lymphocytes associated with moderate exercise [35] or an increase of MN as a consequence of the lack of regular physical exercise (<2 times/week) [72]. On the contrary, excessive physical exercise was associated with an increase of MN frequency [73].

Regarding the relationship between diet and genotoxic DNA damage, numerous studies took into consideration the effect of individual nutrients on MN formation, demonstrating that antioxidants and vitamins lead to a reduction in the frequency of MN [35]; other studies considered the combination of nutrients and the dietary models, as in Villarini et al. [64], who highlighted an increase of MN in the buccal mucosa cells of Italian children aged 6–8 years with a low adherence to MD.

The present study has various strengths. First, the number of recruited children included in the analysis (n = 462) was high and comparable with other Italian and European studies evaluating the frequency of MN in EBC of school-age children. In addition, a consolidated method was used to detect MN in EBC; this allows for the comparison with other molecular epidemiological studies. Finally, investigation of the role of demographic, socio-economic, and lifestyle factors, as possible modifiers of the effect of air pollution on MN frequency, and strict inclusion (e.g., age, residence) and exclusion (e.g., severe diseases, therapy with antineoplastic agents or radiation therapy, exposure to X-rays, or use of dental braces) criteria allowed us to exclude important confounding factors and to evaluate other possible risk factors for cytogenetic damage.

The results of the present study should be considered in light of some limitations. Firstly, as the recruitment was conducted on a voluntary basis, a selection bias caused by cultural or motivational factors cannot be excluded. Secondly, as the personal, residential, and behavioral factors were detected by the use of self-administered questionnaires, it is possible that they were underestimated or overestimated depending on the subjective perception of the parents. Finally, other factors may have had some effect on the MN frequency, such as exposure to pesticides (both exposure to the use of pesticides in agricultural fields and on fresh food treated with pesticides) or electromagnetic waves.

## 5. Conclusions

This study highlighted that the EBC of children living in a heavily anthropized area showed greater genotoxic damage than children living in an area without significant anthropic activities. Among the investigated variables, environmental exposure seemed to be decisive in increasing the frequency of MN. At the same time, lifestyle can play an important role in modulating genotoxic damage.

MN proved to be a sensitive indicator of early biological effects caused by environmental exposure and could be used to determine the risk levels in different populations. Further studies are needed in order to more deeply investigate the role of factors found to be associated with MN and to evaluate the effect due to components from industrial activity and vehicular traffic on the MN frequency in impacted areas.

## Figures and Tables

**Table 1:** Results of recruitment and questionnaire administration activities.

	Impacted Area	Control Area
Selected schools (n)	4	5
Invited children (n)	432	455
Consents (n)	238	310
Valid questionnaires (n)	222	282
Sampled children (n)	206	256

**Table 2:** Individual characteristics of children recruited in the two areas.

	Impacted Area n (%)	Control Area n (%)	*p*-Value
Males	97 (47.1)	143 (55.9)	0.061
Age			
6 years old	47 (22.8)	71 (27.7)	0.421
7 years old	81 (39.3)	89 (34.8)	
8 years old	78 (37.9)	96 (37.5)	
Weight status			
Underweight	24 (11.7)	22 (8.6)	0.493
Normal weight	130 (63.1)	156 (60.9)	
Overweight (without obese)	33 (16.0)	52 (20.3)	
Obese	19 (9.2)	26 (10.2)	
Respiratory problems^1^	60 (29.1)	52 (20.3)	0.028
Taking medicine^2^	33 (16.0)	32 (12.5)	0.280
Sport (≥3 times/week)	127 (61.7)	136 (53.1)	0.066
Adherence to MD^3^			
High	57 (27.7)	75 (29.3)	0.489
Moderate	78 (37.9)	106 (41.4)	
Low	71 (34.5)	75 (29.3)	

^1^ beyond the common cold; ^2^ in addition to common antibiotics, antipyretics, and anti-inflammatory agents; ^3^ according to KIDMED (Mediterranean Diet Quality Index for children and adolescents) score calculated for each child. MD: Mediterranean diet.

**Table 3:** Characteristics of the children’s parents in the two areas.

	Impacted Area n (%)	Control Area n (%)	*p*-Value
Born in Italy			
Mother	193 (93.7)	221 (86.7)	0.013
Father	202 (98.1)	229 (90.2)	0.001
Parents with a college degree			
Mother	60 (29.1)	49 (19.2)	0.013
Father	45 (21.8)	24 (9.4)	<0.001
Employed parents			
Mother	100 (48.6)	122 (47.8)	0.842
Father	169 (82.0)	220 (86.6)	0.177
Parents’ smoking habits			
Mother	51 (24.8)	35 (13.7)	0.003
Father	65 (31.6)	80 (31.5)	0.990

**Table 4:** Exposure factors related to children’s lifestyle and domestic environment.

	Impacted Area n (%)	Control Area n (%)	*p*-Value
Residence in heavily trafficked streets	77 (37.4)	22 (8.6)	<0.001
School near heavily trafficked streets	76 (36.9)	21 (8.2)	<0.001
Use of fireplaces ^1^	29 (14.3)	89 (35.0)	<0.001
Use of wood/pellet stoves ^1^	16 (7.8)	16 (6.3)	0.415
Gas boiler inside the house	20 (9.7)	32 (12.5)	0.345
Frequent presence in the kitchen during food cooking	40 (19.4)	50 (19.5)	0.976
Cooking on the griddle/barbecue inside the home	138 (67.0)	176 (68.7)	0.687
Living with people smoking at home	49 (23.8)	29 (11.3)	0.001
Consumption of fried foods ^1^	184 (89.3)	236 (92.2)	0.556
Consumption of barbecued foods (wood/charcoal) ^1^	101 (49.0)	142 (55.5)	0.168
Consumption of foods cooked on the griddle ^1^	128 (62.1)	133 (52.0)	0.016
Consumption of toasted bread ^1^	107 (51.9)	119 (46.5)	0.417
Consumption of pizza cooked in a wood oven ^1^	133 (64.6)	199 (77.7)	0.002

^1^ in the month preceding the survey.

**Table 5:** Data on particulate matter (PM) fractions measured in the samples from the two areas. Data are reported as mean value of four samples in the impacted area and five samples in control area.

	Impacted Area			Control Area	*p*-Value
	Brindisi	Torchiarolo	Total		
PM_10_ (µg/m ^3^)	22.7 ± 8.5	21.3 ± 4.8	22.2 ± 6.8	11.6 ± 3.9	<0.001
PM_0.5_ (µg/m ^3^)	8.36 ± 3.36	10.59 ± 2.28	9.25 ± 2.55	3.16 ± 1.40	<0.001

**Table 6:** Micronuclei (MN) positive samples (%) and frequency (‰ ± SD) in EBC (exfoliated buccal cells) of children living in the two areas.

	Impacted Area			Control Area	*p*-Value
	Brindisi	Torchiarolo	Total		
Positive samples (%)	66.4	73.7	68.4	37.1	<0.001 ^1^
Frequency (‰ ± SD)	0.64 ± 0.63	0.70 ± 0.56	0.66 ± 0.61	0.27 ± 0.43	<0.001 ^2^

Differences between the two areas were calculated with ^1^ Chi-square test and ^2^ Kruskal–Wallis H test.

**Table 7:** Odds ratios (OR) and 95% confidence interval (CI) of MN frequency in EBC of children associated with individual or environmental factors.

Independent Variables	Odds Ratio	95% CI	*p*-Value
Sex (male)	0.96	0.63 to 1.45	0.844
Age	1.03	0.80 to 1.32	0.830
Normal weight	0.82	0.54 to 1.25	0.360
Respiratory diseases ^1^	0.94	0.56 to 1.59	0.829
Taking medicine ^2^	1.46	0.78 to 2.74	0.242
Sport	0.64	0.42 to 0.97	0.034
High adherence to MD ^3^	0.50	0.27 to 0.94	0.033
Fresh fruit consumption per day	1.03	0.98 to 1.06	0.071
Father employed	1.15	0.64 to 2.06	0.643
Mother employed	1.38	0.89 to 2.13	0.150
Father with a college degree	1.39	0.74 to 2.61	0.303
Mather with a college degree	0.77	0.44 to 1.33	0.345
At least one parent smoker	1.37	0.81 to 2.30	0.242
Residence in heavily trafficked streets	2.12	1.24 to 3.63	0.006
Days of fireplace use per month ^4^	1.00	0.98 to 1.03	0.735
Days of wood/pellet stove use per month ^4^	1.02	0.97 to 1.06	0.430
Internal gas boiler	1.67	0.86 to 3.27	0.132
Living with people smoking at home	0.84	0.42 to 1.65	0.605
Consumption of fried foods ^4^	0.73	0.35 to 1.51	0.392
Consumption of barbecued foods ^4^	1.57	1.02 to 2.40	0.040
Consumption of foods cooked on the griddle ^4^	1.01	0.66 to 1.55	0.949
Consumption of toasted bread ^4^	1.13	0.74 to 1.72	0.580
Consumption of pizza cooked in a wood oven ^4^	1.04	0.66 to 1.65	0.871
PM_0.5_	1.25	1.16 to 1.34	<0.001

^1^ beyond the common cold; ^2^ in addition to common antibiotics, antipyretics, and anti-inflammatory agents; ^3^ according to KIDMED score; ^4^ in the month preceding the survey.

## Supplementary Materials

The following are available online at https://www.mdpi.com/1660-4601/17/4/1208/s1, Document S1: Questionnaire for parents in original (Italian) and English language.
