# Supplementary material for: Micronucleus Frequency in Exfoliated Buccal Cells of Children Living in an Industrialized Area of Apulia (Italy)
Source: Int J Environ Res Public Health. 2020 Feb 13;17(4):1208. doi: 10.3390/ijerph17041208 (PMC7068596; doi:10.3390/ijerph17041208)
Supplement: Supplementary file 1 [file ijerph-17-01208-s001.pdf]

UNIVERSITÀ DEL SALENTO

Campo riservato ai ricercatori. Non compilare.

CODICE QUESTIONARIO |__|__|__|__|

ALTEZZA DE BAMBINO (cm) |__|__|__|

PESO DEL BAMBINO (kg) |__|__|,|__|

QUESTIONARIO PER I GENITORI

DATA DI COMPILAZIONE DEL QUESTIONARIO |__|__| GIORNO |__|__| MESE |__|__|__|__| ANNO

DATA DI NASCITA DI SUO FIGLIO |__|__| GIORNO |__|__| MESE |__|__|__|__| ANNO

SESSO DEL BAMBINO ☐ MASCHIO ☐ FEMMINA

NAZIONE DI NASCITA DI SUO FIGLIO ______________________________

COMUNE DI RESIDENZA DI SUO FIGLIO ______________________________

SCUOLA FREQUENTATA DA SUO FIGLIO ______________________________

CLASSE FREQUENTATA ______________________________

CRITERI DI INCLUSIONE ALLO STUDIO

Suo figlio ha già compiuto 9 anni?
○ SI ○ NO

Negli ultimi 12 mesi suo figlio è stato sottoposto a radioterapia o chemioterapia?
○ SI ○ NO

Negli ultimi 12 mesi suo figlio ha avuto un tumore maligno?
○ SI ○ NO

Nell'ultimo mese suo figlio è stato sottoposto a radiografie?
○ SI ○ NO

Suo figlio ha malattie genetiche (es. Sindrome di Down)?
○ SI ○ NO

Suo figlio utilizza un apparecchio ortodontico?
○ SI ○ NO

Se ha risposto SI anche ad una sola di queste domande suo figlio non possiede i requisiti per partecipare allo studio. Per lei il questionario finisce qui.
La ringraziamo per la gentile partecipazione.

ABITAZIONE DEL FIGLIO

1. Come giudica il traffico nella zona dove si trova l'abitazione di suo figlio?
   - O Scarso o assente
   - O Moderato
   - O Intenso e intermittente
   - O Intenso e continuo

2. Il riscaldamento della sua abitazione è a:
   - O legna o pellet
   - O carbone
   - O gasolio o kerosene
   - O gas
   - O elettricità
   - O altro ______________________________

3. In casa sono presenti delle stufe?
   - O SI
   - O NO

   se **SI**, nell'ultimo mese quante volte ha usato la stufa (per riscaldare o per cucinare)?
   - O Mai
   - O Numero di volte |__|__|

   se **SI**, he tipo di combustibile viene usato per la stufa?
   - O legna o pellet
   - O gas
   - O elettricità
   - O altro ___________________________

4. E' presente un caminetto in casa?
   - O SI
   - O NO

   se **SI**, nell'ultimo mese quante volte è stato usato il caminetto?
   - O Mai
   - O Numero di volte |__ |__ |

   se **SI**, che tipo di combustibile viene usato per il caminetto?
   - O legna o pellet
   - O carbonella
   - O altro _________________________

5. Quale combustibile viene generalmente usato per cucinare?
   - O elettricità
   - O gas
   - O legna o carbone
   - O altro ______________________________

6. C'è uno scaldabagno o boiler a gas all'interno dei locali dell'abitazione?
   - O SI
   - O NO

SCUOLA FREQUENTATA DA SUO FIGLIO

7. Come giudica il traffico nella zona dove si trova la scuola di suo figlio?
   - O Scarso o assente
   - O Moderato
   - O Intenso e intermittente (ad es. solo nelle ore di punta)
   - O Intenso e continuo, per buona parte del giorno

STATO DI SALUTE DI SUO FIGLIO

8. Suo figlio ha un grave problema di salute?
   - O SI
   - O NO

   se SI, quale? ___________________________

9. Suo figlio assume o ha assunto farmaci negli ultimi sei mesi (al di fuori dei comuni farmaci antibiotici, antipiretici e antiinfiammatori)?
   - O SI
   - O NO

10. Suo figlio soffre o ha sofferto negli ultimi 12 mesi di disturbi respiratori al di fuori dei comuni raffreddori?
    - O SI
    - O NO

    se **SI**, indicare quale/quali?
    - O attacchi di asma
    - O fischi e sibili
    - O tosse
    - O catarro
    - O disturbi nasali persistenti e/o ricorrenti (muco nasale, naso chiuso)
    - O raffreddore allergico

ABITUDINI DI VITA DI SUO FIGLIO

11. Suo figlio pratica regolarmente attività sportiva più volte alla settimana? (sport, ginnastica, nuoto, danza o altre attività fisiche svolte in palestra o all'aperto)
   - O SI
   - O NO

   se **SI**, pratica attività sportiva all'aperto?
   - O SI
   - O NO

   Se **SI**, frequenta una piscina più volte alla settimana?
   - O SI
   - O NO

12. Quante ore al giorno, suo figlio, mediamente, gioca all'aria aperta, in questa stagione?
   - O meno di 1 ora
   - O da 1 a 2 ore
   - O da 2 a 3 ore
   - O da 3 a 4 ore
   - O più di 4 ore

13. Attualmente qualcuno fuma nell'abitazione dove vive suo figlio?
   - O SI
   - O NO

14. Capita qualche volta che suo figlio si trovi con persone che fumano in ambienti chiusi (in auto, in casa, ecc.)?
   - O SI
   - O NO

15. Qualcuno della famiglia usa solventi per hobby all'interno dell'abitazione (colle, vernici, colori, ecc.)?
   - O SI
   - O NO

16. Suo figlio sta in cucina durante la cottura dei cibi?
   - O mai
   - O qualche volta
   - O spesso/sempre

17. Indicativamente, quante volte al mese vengono cotti in casa cibi alla piastra (bistecchiera o superficie rovente) o alla griglia (legna, carbonella)?

   O mai oppure O N. volte/mese |___|___|

18. Suo figlio frequenta fast food più di una volta a settimana?
   - O SI
   - O NO

19. Nell'ultimo mese, può indicare la frequenza con cui suo figlio ha mangiato i seguenti alimenti? (escludendo il cibo consumato a scuola)

cibi fritti	O mai
	O N. volte/mese |___|___|
cibi alla griglia*	O mai
	O N. volte/mese |___|___|
cibi alla piastra**	O mai
	O N. volte/mese |___|___|
pane tostato	O mai
	O N. volte/mese |___|___|
pizza cotta a legna	O mai
	O N. volte/mese |___|___|

* cibi cucinati su legna o carbonella, non su griglia elettrica
** bistecchiera o superficie rovente

20. Suo figlio salta la colazione?
   - O mai
   - O qualche volta
   - O spesso/sempre

21. Suo figlio consuma prodotti caseari (latte o yogurt) a colazione?
   - O SI
   - O NO

22. Suo figlio consuma abitualmente cereali o pane a colazione?
   - O SI
   - O NO

23. Suo figlio consuma prodotti industriali come biscotti, merendine o altri dolci, a colazione?
   - O SI
   - O NO

LA MAMMA

24. In quale nazione è nata la mamma del bambino?

____

25. Qual è il titolo di studio della mamma?

- O Non ha titoli di studio
- O Licenza elementare
- O Licenza media
- O Diploma di scuola media superiore
- O Laurea

26. La mamma fuma sigarette?

O SI O NO

27. La mamma svolge attività lavorativa (autonoma, dipendente, imprenditrice)?

O SI O NO

se **SI**, quale tra le seguenti categorie professionali, comprende il lavoro della mamma?

- O dirigente
- O direttivo/quadro (include docenti di scuola secondaria)
- O tecnica o impiegata ad alta/media qualificazione (ad es., geometri, periti tecnici, impiegate amministrative, infermiere, docenti di scuole materne ed elementari)
- O impiegata esecutiva (es. segretarie, personale di sportello)
- O capo operaia, operaia specializzata
- O operaia generica, lavoratrice manuale, personale ausiliario (ad es. braccianti, usciere, commesse, cameriere)
- O imprenditrice
- O libera professionista (include persone con contratti di collaborazione coordinata e continuativa o di collaborazione occasionale)
- O lavoratrice in proprio (include commercianti, artigiane, coadiuvanti e socie di cooperativa)

se **NO**, indichi, l'attuale condizione della mamma:

- O Disoccupata in cerca di nuova occupazione
- O In cerca di prima occupazione
- O Casalinga
- O Studentessa
- O Pensionata
- O In altra condizione

IL PAPÀ

28. In quale nazione è nato il papà del bambino?

____

29. Qual è il titolo di studio del papà?

- O Non ha titoli di studio
- O Licenza elementare
- O Licenza media
- O Diploma di scuola media superiore
- O Laurea

30. Il papà fuma sigarette?

O SI O NO

31. Il papà svolge attività lavorativa (autonoma, dipendente, imprenditore)?

O SI O NO

se **SI**, quale tra le seguenti categorie professionali, comprende il lavoro del papà?

- O dirigente
- O direttivo/quadro/funzionaria (include docenti di scuola secondaria)
- O tecnico o impiegato ad alta/media qualificazione (ad es. geometri, periti tecnici, impiegati amministrativi, infermieri, docenti di scuole materne ed elementari)
- O impiegato esecutivo (es. segretari, personale di sportello)
- O capo operaio, operaio specializzato
- O operaio generico, lavoratore manuale, personale ausiliario (ad es. braccianti, usciere, commessi, camerieri)
- O imprenditore
- O libero professionista (include persone con contratti di collaborazione coordinata e continuativa o di collaborazione occasionale)
- O lavoratore in proprio (include commercianti, artigiani, coadiuvanti e soci di cooperativa)

se **NO**, indichi, l'attuale condizione del papà:

- O Disoccupato in cerca di nuova occupazione
- O In cerca di prima occupazione
- O Casalingo
- O Studente
- O Pensionato
- O In altra condizione

FREQUENZA DI CONSUMO DEI PRINCIPALI ALIMENTI

		Mai	Meno di 1 volta a settimana	1-2 volte a settimana	3-4 volte a settimana	5-6 volte a settimana	1 volta al giorno	2 volte al giorno	3 o più volte al giorno
	**PANE, PASTA, CEREALI**								
1	Cereali a colazione	○	○	○	○	○	○	○	○
2	Pane/panini	○	○	○	○	○	○	○	○
3	Crackers, grissini, fette biscottate (sostitutivi del pane)	○	○	○	○	○	○	○	○
4	Pasta asciutta	○	○	○	○	○	○	○	○
5	Riso/Risotti	○	○	○	○	○	○	○	○
6	Minestra in brodo (pastina)	○	○	○	○	○	○	○	○
7	Tortellini, ravioli, agnolotti	○	○	○	○	○	○	○	○
8	Pasta al forno (lasagne, cannelloni, ecc.)	○	○	○	○	○	○	○	○
9	Zuppe d'orzo o farro	○	○	○	○	○	○	○	○
10	Polenta	○	○	○	○	○	○	○	○
11	Pizza	○	○	○	○	○	○	○	○
12	Focaccia al forno	○	○	○	○	○	○	○	○
**13**	**LEGUMI**	○	○	○	○	○	○	○	○
	**VERDURE**								
14	Minestroni o passati di sole verdure	○	○	○	○	○	○	○	○
15	Patate	○	○	○	○	○	○	○	○
16	Verdure cotte (spinaci, bietole, cavoli, broccoli, asparagi, melanzane, zucchine, zucca, carciofi, fagiolini, ecc)	○	○	○	○	○	○	○	○
17	Verdure crude (insalate, pomodori, finocchi, carote, ecc.)	○	○	○	○	○	○	○	○
18	Funghi	○	○	○	○	○	○	○	○
19	Olive verdi o nere	○	○	○	○	○	○	○	○
**20**	**FRUTTA FRESCA (comprese le spremute di frutta) NO SUCCHI DI FRUTTA CONFEZIONATI**	○	○	○	○	○	○	○	○
**21**	**FRUTTA SECCA (noci, mandorle, nocciole)**	○	○	○	○	○	○	○	○
	**CARNI**								
22	Vitello, manzo (escluse salsicce o hamburger)	○	○	○	○	○	○	○	○
23	Maiale (escluse salsicce o hamburger)	○	○	○	○	○	○	○	○
24	Salsiccia, hamburger	○	○	○	○	○	○	○	○
25	Capretto, agnello, coniglio	○	○	○	○	○	○	○	○
26	Pollo, tacchino	○	○	○	○	○	○	○	○
27	Carne in scatola	○	○	○	○	○	○	○	○
28	Fegato di vitello o maiale	○	○	○	○	○	○	○	○
29	Frattaglie	○	○	○	○	○	○	○	○
	**INSACCATI E PROSCIUTTI**								
30	Wurstel, salami, mortadella	○	○	○	○	○	○	○	○
31	Prosciutto cotto, pancetta, speck, prosciutto crudo	○	○	○	○	○	○	○	○
	**PESCE E FRUTTI DI MARE**								
32	Frutti di mare (molluschi bivalvi, polpo, seppie, calamari, gamberi, aragosta, ecc.)	○	○	○	○	○	○	○	○
33	Tonno in scatola	○	○	○	○	○	○	○	○
34	Bastoncini di pesce	○	○	○	○	○	○	○	○

		Mai	Meno di 1 volta a settimana	1-2 volte a settimana	3-4 volte a settimana	5-6 volte a settimana	1 volta al giorno	2 volte al giorno	3 o più volte al giorno
35	Pesce (es. dentice, sogliola, orata, branzino, cefalo, merluzzo, tonno, pesce spada, sgombro, alici, sardine, salmone, trota, ecc)	○	○	○	○	○	○	○	○
	**VARIE**								
36	Uova (incluse frittate e omelette)	○	○	○	○	○	○	○	○
37	Maionese	○	○	○	○	○	○	○	○
	**LATTE, LATTICINI, FORMAGGI**								
38	Latte intero	○	○	○	○	○	○	○	○
39	Latte parzialmente scremato	○	○	○	○	○	○	○	○
40	Ricotta	○	○	○	○	○	○	○	○
41	Formaggi grattugiati	○	○	○	○	○	○	○	○
42	Mozzarella, fiordilatte, giuncata	○	○	○	○	○	○	○	○
43	Formaggi vari, a pezzi o a fette (caciocavallo, emmenthal, fontina, provolone, parmigiano, stracchino, brie, gorgonzola, scamorza, galbanino)	○	○	○	○	○	○	○	○
44	Formaggi fusi (formaggini, formaggi spalmabili, sottilette)	○	○	○	○	○	○	○	○
45	Mascarpone, panna	○	○	○	○	○	○	○	○
46	Yogurt magro (da bere o al cucchiaio)	○	○	○	○	○	○	○	○
47	Yogurt intero (da bere o al cucc.)	○	○	○	○	○	○	○	○
	**BISCOTTI, DOLCI, DESSERT, SNACK**								
48	Biscotti secchi (frollini, pavesini, granturchese, novellino, ecc)	○	○	○	○	○	○	○	○
49	Biscotti farciti (tipo oro ciok, ringo, wafer, ecc)	○	○	○	○	○	○	○	○
50	Dolci non farciti (Focacce dolci, brioche, torte senza crema, plum cake, ciambelle, ecc.)	○	○	○	○	○	○	○	○
51	Torte o merendine farcite	○	○	○	○	○	○	○	○
52	Dolci al cucchiaio (tipo tiramisù, panna cotta. ecc.)	○	○	○	○	○	○	○	○
53	Cioccolata e snack a base di cioccolato	○	○	○	○	○	○	○	○
54	Caramelle	○	○	○	○	○	○	○	○
55	Gelati alla frutta	○	○	○	○	○	○	○	○
56	Gelati alla panna/crema	○	○	○	○	○	○	○	○
57	Snack salati (patatine, pizzette, salatini, ecc)	○	○	○	○	○	○	○	○

	**BEVANDE**									
		**Mai**	**1 volta al mese o meno**	**Meno di una volta a settimana**	**1-2 volte a settimana**	**Meno di 1 volta al dì**	**1 volta al dì**	**2-3 volte al dì**	**4-5 volte al dì**	**6 o più volte al dì**
58	Coca cola	○	○	○	○	○	○	○	○	○
59	Aranciata o altre bevande gassate	○	○	○	○	○	○	○	○	○
60	Succo di frutta (confezionato)	○	○	○	○	○	○	○	○	○
61	Tazza d’orzo	○	○	○	○	○	○	○	○	○
62	Tazza di tè	○	○	○	○	○	○	○	○	○
63	1 cucchiaino di zucchero (nel tè, nel latte, etc.)	○	○	○	○	○	○	○	○	○
64	1 cucchiaio di miele (nel tè, nel latte, etc.)	○	○	○	○	○	○	○	○	○

	TIPOLOGIA DI COTTURA/PREPARAZIONE							
		**Mai**	**Meno di 1 volta a settimana**	**1-2 volte a settimana**	**3-4 volte a settimana**	**5-6 volte a settimana**	**1 volta al giorno**	**2 volte al giorno**
65	Fritture	○	○	○	○	○	○	○
66	Condimenti sulla pasta	○	○	○	○	○	○	○
67	Cibi cotti al forno	○	○	○	○	○	○	○
68	Cibi cotti in padella	○	○	○	○	○	○	○
69	Verdure crude	○	○	○	○	○	○	○
70	Verdure lesse	○	○	○	○	○	○	○
71	Dolci fatti in casa	○	○	○	○	○	○	○

Indichi il tipo di grasso utilizzato di solito. **SOLO UN TIPO DI GRASSO PER CIASCUN TIPO DI COTTURA**

		**Burro**	**Margarina**	**Olio di oliva**	**Olio di arachidi**	**Altri oli di semi**
72	Fritture	○	○	○	○	○
73	Condimenti sulla pasta	○	○	○	○	○
74	Cibi cotti al forno	○	○	○	○	○
75	Cibi cotti in padella	○	○	○	○	○
76	Verdure crude	○	○	○	○	○
77	Verdure lesse	○	○	○	○	○
78	Dolci fatti in casa	○	○	○	○	○

UNIVERSITÀ DEL SALENTO

Field reserved for researchers. Do not fill in.

QUESTIONNAIRE CODE	|__|__|__|__|
CHILD'S HEIGHT (cm)	|__|__|__|
CHILD'S WEIGHT (kg)	|__|__|,|__|

QUESTIONNAIRE FOR PARENTS

DATE OF COMPLETION OF THE QUESTIONNAIRE |__|__| DAY |__|__| MONTH |__|__|__|__| YEAR

CHILD'S DATE OF BIRTH |__|__| DAY |__|__| MONTH |__|__|__|__| YEAR

CHILD'S GENDER ☐ MALE ☐ FEMALE

CHILD'S COUNTRY OF BIRTH ____

CHILD'S CITY OF RESIDENCE ____

CHILD'S SCHOOL ____

CLASS ____

INCLUSION CRITERIA OF THE STUDY

Is your child already 9 years old?
O YES O NO

Has your child undergone chemotherapy or radiotherapy in the last 12 months?
O YES O NO

Has your child had a malignancy in the last 12 months?
O YES O NO

Has your child been exposed to x-rays in the last month?
O YES O NO

Does your child suffer from genetic diseases (i.e. Down syndrome)?
O YES O NO

Does your child use dental braces?
O YES O NO

If you answered <u>YES</u> to even <u>one</u> of these questions the questionnaire ends here.
Thanks for your kind cooperation.

CHILD'S HOME

1. How do you rate the traffic in the area where your child's home is located?
   - O Low or nil
   - O Moderate
   - O Heavy and intermittent
   - O Heavy and smooth
2. The fuel for heating your home is:
   - O Wood or pellets
   - O Coal
   - O Diesel
   - O Gas
   - O Electricity
   - O Other ______________________
3. Is there a stove in your house?
   - O YES
   - O NO

   if **YES**, how many times have you used the stove in the last month (for heating or cooking)?
   - O Never
   - O Number of times |__|__|

   if **YES**, what type of fuel is used for the stove?
   - O Wood or pellets
   - O Gas
   - O Electricity
   - O Other ______________________
4. Is there a fireplace in your house?
   - O YES
   - O NO

   if **YES**, how many times has the fireplace been used in the last month?
   - O Never
   - O Number of times |__ |__ |

   if **YES**, what type of fuel is used for the fireplace?
   - O Wood or pellets
   - O Coal
   - O Other ______________________
5. What fuel is generally used for cooking?
   - O Electricity
   - O Gas
   - O Wood or coal
   - O Other ______________________
6. Is there a gas boiler inside your house?
   - O YES
   - O NO

CHILD'S SCHOOL

7. How do you rate the traffic in the area where your child's school is located?
   - O Low or nil
   - O Moderate
   - O Heavy and intermittent
   - O Heavy and smooth

HEALTH STATUS OF YOUR CHILD

8. Does your child have a serious health problem?
   - O YES
   - O NO

   if **YES**, which one? ______________________
9. Does your child take medicine in addition to common remedies (such as antibiotics, antipyretics and anti-inflammatory agents) in the last six months?
   - O YES
   - O NO
10. Has your child suffered from respiratory problems beyond the common cold in the last 12 months?
    - O YES
    - O NO

    if **YES**, which ones?
    - O Asthma
    - O Hisses
    - O Cough
    - O Pleghm
    - O Persistent and / or recurrent nasal upset (nasal discharge, stuffy nose)
    - O Allergic cold

CHILD'S LIFESTYLE

11. Does your child regularly play sports several times a week? (swimming, dancing or other physical activities carried out in the gym or outdoors)
    - O YES
    - O NO

    if **YES**, does he/she play outdoor sports?
    - O YES
    - O NO

    if **YES**, does he/she go to a swimming pool several times a week?
    - O YES
    - O NO

12. How many hours per day, on average, does your child play outdoor in this season?
    - O less than 1 hour
    - O 1 to 2 hours
    - O 2 to 3 hours
    - O 3 to 4 hours
    - O more than 4 hours

13. Does anyone currently smoke in the house where your child lives?
    - O YES
    - O NO

14. Is it possible that sometimes your child is with people who smoke indoors (in the car, inside the home, etc.)?
    - O YES
    - O NO

15. Does anyone in the family use solvents for hobbies inside the home (glues, paints, colors, etc.)?
    - O YES
    - O NO

16. Does your child stay in the kitchen while cooking food?
    - O Never
    - O Sometimes
    - O Often/always

17. How many times a month are foods cooked on the griddle (hot surface) or grill (wood, charcoal) inside the home?

    O Never or O times/month |___|___|

18. Does your child go to fast food restaurants more than once a week?
    - O YES
    - O NO

19. In the last month, how often your child has eaten the following foods? (excluding food consumed at school)

Fried foods	O never
	O times/month |___|___|
barbecued foods*	O never
	O times/month |___|___|
grilled foods**	O never
	O times/month |___|___|
toasted bread	O never
	O times/month |___|___|
wood oven pizza	O never
	O times/month |___|___|

* foods cooked on wood or charcoal, not on electric grill
** grill pan or hot surface

20. Does your child skip breakfast?
    - O Never
    - O Sometimes
    - O Often/always

21. Does your child have dairy products (milk or yogurt) for breakfast?
    - O YES
    - O NO

22. Does your child have cereals or grains (bread, etc.) for breakfast?
    - O YES
    - O NO

23. Does your child have commercially baked goods or pastries for breakfast?
    - O YES
    - O NO

MOTHER

24. In which country was the child's mother born?

____

25. What is mother's educational qualification?

- ○ None
- ○ Primary school diploma
- ○ Middle school diploma
- ○ High school diploma
- ○ College degree

26. Does child's mother smoke cigarettes?

○ YES ○ NO

27. Does child's mother work?

○ YES ○ NO

if **YES**, in which of the following professional categories, is the mother's job included?

- ○ Senior manager
- ○ Chief executive (including secondary education teachers)
- ○ Skilled technician or employee (i.e. surveyors, technical experts, administrative employees, nurses, primary school teachers)
- ○ Executive employee (i.e. secretary)
- ○ Chief or senior worker
- ○ General worker (i.e. day laborer, shop assistant, waiter)
- ○ Business owner
- ○ Professional
- ○ Self employed (including merchants, craftsmen, assistants and cooperative members)

if **NO**, indicate the current condition of the mother:

- ○ Unemployed, job seeker
- ○ Seeking the first employment
- ○ Housewife
- ○ Student
- ○ Retired
- ○ Other condition

FATHER

28. In which country was the child's father born?

____

29. What is father's educational qualification?

- ○ None
- ○ Primary school diploma
- ○ Middle school diploma
- ○ High school diploma
- ○ College degree

30. Does child's father smoke cigarettes?

○ YES ○ NO

31. Does child's father work?

○ YES ○ NO

if **YES**, in which of the following professional categories, is the father's job included?

- ○ Senior manager
- ○ Chief executive (including secondary education teachers)
- ○ Skilled technician or employee (i.e. surveyors, technical experts, administrative employees, nurses, primary school teachers)
- ○ Executive employee (i.e. secretary)
- ○ Chief or senior worker
- ○ General worker (i.e. day laborer, shop assistant, waiter)
- ○ Business owner
- ○ Professional
- ○ Self employed (including merchants, craftsmen, assistants and cooperative members)

if **NO**, indicate the current condition of the father:

- ○ Unemployed, job seeker
- ○ Seeking the first employment
- ○ Househusband
- ○ Student
- ○ Retired
- ○ Other condition

FREQUENCY OF CONSUMPTION OF THE MAIN FOODS

		Never	Less than once a week	1-2 times a week	3-4 times a week	5-6 times a week	Once a day	Twice a day	3 or more times a day
	**BREAD, PASTA, CEREALS**								
1	Cereals for breakfast	○	○	○	○	○	○	○	○
2	Bread	○	○	○	○	○	○	○	○
3	Crackers, breadsticks, rusks (bread substitutes)	○	○	○	○	○	○	○	○
4	Pasta	○	○	○	○	○	○	○	○
5	Rice/Risotto	○	○	○	○	○	○	○	○
6	Soups in broth	○	○	○	○	○	○	○	○
7	Tortellini, ravioli, agnolotti (stuffed pasta)	○	○	○	○	○	○	○	○
8	Baked pasta (i.e. lasagna, cannelloni)	○	○	○	○	○	○	○	○
9	Barley or spelled soups	○	○	○	○	○	○	○	○
10	Polenta	○	○	○	○	○	○	○	○
11	Pizza	○	○	○	○	○	○	○	○
12	Baked focaccia							○	
**13**	**LEGUMES**	○	○	○	○	○	○	○	○
	**VEGETABLES**								
14	Minestrone or pureed vegetables	○	○	○	○	○	○	○	○
15	Potatoes	○	○	○	○	○	○	○	○
16	Cooked vegetables (spinach, chard, cabbage, broccoli, asparagus, eggplant, zucchini, pumpkin, artichokes, green beans, etc.)	○	○	○	○	○	○	○	○
17	Raw vegetables (salad, tomatoes, fennel, carrots, etc.)	○	○	○	○	○	○	○	○
18	Mushrooms	○	○	○	○	○	○	○	○
19	Olives	○	○	○	○	○	○	○	○
**20**	**FRESH FRUIT (including fruit juices) NO PACKED FRUIT JUICES**	○	○	○	○	○	○	○	○
**21**	**DRIED FRUIT (walnuts, almonds, hazelnuts)**	○	○	○	○	○	○	○	○
	**MEAT**								
22	Veal, beef (excluding sausage or hamburger)	○	○	○	○	○	○	○	○
23	Pork (excluding sausage or hamburger)	○	○	○	○	○	○	○	○
24	Sausage, hamburger	○	○	○	○	○	○	○	○
25	Goat, lamb, rabbit	○	○	○	○	○	○	○	○
26	Chicken, turkey	○	○	○	○	○	○	○	○
27	Canned meat	○	○	○	○	○	○	○	○
28	Veal or pork liver	○	○	○	○	○	○	○	○
29	Offal	○	○	○	○	○	○	○	○
	**PROCESSED MEAT AND HAM**								
30	Frankfurters, salami, mortadella	○	○	○	○	○	○	○	○
31	Baked ham, bacon, speck, dry cured ham	○	○	○	○	○	○	○	○
	**FISH AND SHELLFISH**								
32	Shellfish (bivalve mollusks, octopus, cuttlefish, squid, prawns, lobster, etc.)	○	○	○	○	○	○	○	○
33	Canned tuna	○	○	○	○	○	○	○	○

		Never	Less than once a week	1-2 times a week	3-4 times a week	5-6 times a week	Once a day	Twice a day	3 or more times a day
34	Fish sticks	○	○	○	○	○	○	○	○
35	Fish (i.e. snapper, sole, sea bream, sea bass, mullet, cod, tuna, swordfish, mackerel, anchovies, sardines, salmon, trout etc.)	○	○	○	○	○	○	○	○
	**OTHER**								
36	Eggs (including omelet)	○	○	○	○	○	○	○	○
37	Mayonnaise	○	○	○	○	○	○	○	○
	**MILK, DAIRY PRODUCTS, CHEESES**								
38	Full-fat milk	○	○	○	○	○	○	○	○
39	Low-fat milk	○	○	○	○	○	○	○	○
40	Ricotta cheese	○	○	○	○	○	○	○	○
41	Grated cheese	○	○	○	○	○	○	○	○
42	Mozzarella	○	○	○	○	○	○	○	○
43	Cheese in pieces (caciocavallo, emmenthal, fontina, provolone, parmesan, stracchino, brie, gorgonzola, scamorza)	○	○	○	○	○	○	○	○
44	Processed cheese (sottilette, spreadable cheese, etc.)	○	○	○	○	○	○	○	○
45	Mascarpone cheese, cooking cream	○	○	○	○	○	○	○	○
46	Low-fat yogurt	○	○	○	○	○	○	○	○
47	Full-fat yogurt	○	○	○	○	○	○	○	○
	**COOKIES, CAKES, DESSERTS, SNACKS**								
48	Dry cookies	○	○	○	○	○	○	○	○
49	Stuffed cookies	○	○	○	○	○	○	○	○
50	Unfilled cakes (bun, brioche, plum cake, etc.)	○	○	○	○	○	○	○	○
51	Stuffed cakes or snacks	○	○	○	○	○	○	○	○
52	Soft desserts, milk puddings and custards (i.e. tiramisu, panna cotta)	○	○	○	○	○	○	○	○
53	Chocolate and chocolate snacks	○	○	○	○	○	○	○	○
54	Candies	○	○	○	○	○	○	○	○
55	Fruit ice cream	○	○	○	○	○	○	○	○
56	Ice cream	○	○	○	○	○	○	○	○
57	Savory snacks (chips, pretzel, etc.)	○	○	○	○	○	○	○	○

	**DRINKS**									
		**Never**	**Once a month or less**	**Less than once a week**	**1-2 times a week**	**Less than once a day**	**Once a day**	**2-3 times a day**	**4-5 times a day**	**6 or more times a day**
58	Coca cola	○	○	○	○	○	○	○	○	○
59	Orange soda or other carbonated drinks	○	○	○	○	○	○	○	○	○
60	Fruit juice (packed)	○	○	○	○	○	○	○	○	○
61	A cup of barley coffee	○	○	○	○	○	○	○	○	○
62	A cup of tea	○	○	○	○	○	○	○	○	○
63	1 teaspoon sugar (in tea, in milk, etc.)	○	○	○	○	○	○	○	○	○
64	1 teaspoon honey (in tea, in milk, etc.)	○	○	○	○	○	○	○	○	○

	PREPARATION/COOKING METHODS							
		**Never**	**Less than once a week**	**1-2 times a week**	**3-4 times a week**	**5-6 times a week**	**Once a day**	**Twice a day**
65	Frying	○	○	○	○	○	○	○
66	Pasta seasonings	○	○	○	○	○	○	○
67	Baked foods	○	○	○	○	○	○	○
68	Pan food cooked	○	○	○	○	○	○	○
69	Raw vegetables	○	○	○	○	○	○	○
70	Boiled vegetables	○	○	○	○	○	○	○
71	Homemade sweets	○	○	○	○	○	○	○

Please, indicate the type of fat usually used. **ONLY ONE TYPE OF FAT FOR EACH TYPE OF COOKING.**

		**Butter**	**Margarine**	**Olive oil**	**Peanut oil**	**Other seed oils**
72	Frying	○	○	○	○	○
73	Pasta seasonings	○	○	○	○	○
74	Baked foods	○	○	○	○	○
75	Pan food cooked	○	○	○	○	○
76	Raw vegetables	○	○	○	○	○
77	Boiled vegetables	○	○	○	○	○
78	Homemade sweets	○	○	○	○	○
